# Clinical outcomes of frozen-thawed blastocysts with twice noninvasive chromosome screenings

**DOI:** 10.3389/fendo.2025.1699690

**Published:** 2025-10-30

**Authors:** Yu Qiao, Shuangshuang Geng, Bin Zhang, Fanyu Meng, Weimin Yang, Chenyi Wang, Yaxin Yao, Dunmei Zhao, Sijia Lu, Liyi Cai, Kai Deng

**Affiliations:** ^1^ Reproductive Medicine Department, Hebei Maternity Hospital, Shijiazhuang, China; ^2^ Shi Jiazhuang Technology Innovation Center of Precision Prevention and Control of Birth Defects, Shijiazhuang, China; ^3^ Department of Clinical Research, Yikon Genomics Co., Ltd., Suzhou, China; ^4^ School of Biomedical Engineering, Hubei University of Medicine, Shiyan, China

**Keywords:** noninvasive chromosome screening, frozen-thawed embryos, clinical pregnancy outcome, blastocyst transfer, NICS detection failure

## Abstract

**Research Question:**

Does the double freeze–thaw procedure affect embryo quality or clinical outcomes for patients?

**Design:**

A retrospective study was conducted on patients undergoing noninvasive chromosome screening (NICS) from March 2018 to April 2024. Patients were divided into two groups: (1) the double freeze-thaw group, whose cryopreserved blastocysts underwent a second NICS after thawing because the first NICS test failed, and (2) the single freeze-thaw group, whose blastocysts were successfully analysed in the first NICS. The clinical outcomes included the detection success rate of NICS via the analysis of thawing culture medium and the live birth rate.

**Results:**

A total of 275 patients and 1, 443 embryos were included, with a NICS detection failure rate of 6.7% (96/1, 443). 87 were re-analysed after a second NICS using their thawed culture medium; 57.4% (50/87) of these re-tested embryos were classified as grade A or B. Fifty-two embryos were thawed and transferred in the double freeze-thaw group. Compared with the morphological grading before the first freezing, the ICM grade of two embryos decreased from A to B, and the TE grade of two embryos decreased from B to C before the second freezing. The results showed that there were still no significant differences in the clinical pregnancy rate (56.52% vs. 57.14%, adjusted p=0.785), early miscarriage rate (21.98% vs. 25.00%, adjusted p=0.528), ongoing pregnancy rate (44.10% vs. 42.86%, adjusted p=0.516), and live birth rate (42.86% vs. 42.86%, adjusted p=0.736) in single freeze–thaw group and double freeze–thaw group.

**Conclusions:**

Comparable clinical outcomes were achieved by re-applying NICS using the thawing culture medium compared to the single freeze-thaw group.

## Introduction

Noninvasive chromosome screening (NICS) is a novel method for preimplantation chromosomal screening of embryos (1). This technique utilizes the blastocyst bathing culture medium as the sample for testing and combines whole-genome amplification (WGA) with next generation sequencing (NGS). By analyzing the chromosomal status of cell-free DNA in the embryo culture medium, NICS provides a comprehensive assessment of the embryo’s chromosomal status, thereby providing preferences in selecting the most viable embryos for transferring (2). In recent years, multiple clinical studies have demonstrated that the accuracy of NICS can be comparable to that of preimplantation genetic testing for aneuploidy (PGT-A) (3, 4). For patients with recurrent miscarriages, recurrent implantation failure, and advanced maternal ages, NICS can improve clinical outcomes (2, 5). However, NICS technology has limitations, particularly regarding to detection success rates as referring to straightforward CNV result or non-N/A per say, which vary across studies (77.3%–100%) (6–9). Multiple factors influence the detection success rate of NICS, including embryo morphology, expansion degree and the sampling time. Because embryos that fail NICS do not generate a CNV profile, clinicians remain cautious about transferring them.

For embryos that initially failed PGT-A, studies have shown that thawing the embryos, performing a second biopsy, and selecting embryos for transfer based on the second PGT-A results can maintain a satisfactory live birth rate (10, 11). However, a retrospective cohort study indicates that double biopsies may negatively impact clinical outcomes, as the clinical pregnancy rate of those blastocysts experienced double biopsies is lower compared to that obtained with single biopsy in frozen embryo transfers (12). Therefore, a non-invasive sampling approach is required for the secondary assessment of cryopreserved embryos that fail the initial PGT-A analysis. NICS is a non-invasive sampling method, and previous studies have thawed cryopreserved blastocysts and collected spent culture medium at different time-points for NICS analysis. Frozen embryos were thawed and cultured for 14–24 hours, after which the culture medium was collected for NGS. The detection success rate of NICS was 92.3%–100%, and its accuracy was 87%–100% when using the whole embryo results as the gold standard (13, 14). Kuznyetsov et al. found that using the culture medium of thawed frozen embryos for NICS detection, with the whole embryo as the gold standard, the consistency of NICS was higher than that of TE (96.4% vs. 91.7%) (7). These findings demonstrated the feasibility and potential benefits of using the culture medium of thawed frozen embryos for NICS detection.

However, the culture time after thawing of frozen embryos was relatively long (14–24 hours); currently, no complement achieved along the application of NICS on the bathing time. Moreover, the clinical outcomes of blastocysts transfer after a second freeze-thaw cycle remain unknown. Thus, the clinical application of this approach requires further investigation. This study focused on frozen embryos that failed the initial NICS test and were subsequently re-cultured for 8 hours. We would compare the morphological changes of embryos before and after double freezing, as well as the clinical outcomes in the two groups. It aims to investigate whether embryos that have undergone two freeze-thaw cycles impact clinical outcomes in patients undergoing NICS.

## Methods

### Study design

This single-center retrospective clinical study recruited patients from Hebei Maternity Hospital between March 2018 and April 2024 with signed acknowledgement letter. All pregnancy outcomes was collected in May 2025. This study was reviewed and approved by the Medical Science Research Ethics Committee of Hebei Reproductive Maternity Hospital (ID: 20240002).

### Participants

The study enrolled patients with the application of assisted reproduction as frozen embryo transfer (FET). Inclusion criteria as listed:

1. The female patient was 22–40 years old with BMI locates between 18–25 kg/m^2^;
2. Couples accept NICS testing; and the preference of embryo transfer based on the results of NICS.

Exclusion criteria expelled patients with chromosomal abnormalities, uterine anomalies, and endometrial thickness (<7 mm).;

The culture medium after second thawing was collected for another NICS detection, where transfer was guided by this results. Those embryos were transferred based on the second NICS results when assigned to the double freeze-thaw group as the test group, while rest of embryos who relys on the initial NICS results were included to the single freeze-thaw group where belonging to the conrtrol group.

### Oocytes retrieval and granulosa cell removal

Based on the patient’s specific case, ovulation was stimulated by standard antagonist and progestin primed ovarian stimulation (PPOS), and the dose of gonadotropin was adjusted according to the patient’s ovarian response, hormone level and follicle size. When the follicle diameter and hormone levels reached triggering criteria, patients received either a dose of 5, 000 to 8, 000 IU human chorionic gonadotropin (HCG) or 0.1 mg gonadotropin-releasing hormone agonist (GnRHa) combined with 4, 000 IU HCG. Egg retrieval was performed approximately 37 hours after triggering under the guidance of transvaginal ultrasound. One to two hours after egg retrieval, oocytes were treated with hyaluronidase and blown and washed three times to remove granulosa cells.

### Embryo culture and first sample collection

For intracytoplasmic sperm injection (ICSI) embryo, fertilization was assessed 16–18 hours after ICSI. For conventional *in vitro* fertilization (IVF) embryo, confirmation was performed 18–19 hours after sperm insemination. Two pronuclei and two polar bodies were visualized clearly. On the afternoon of day 2 post-fertilization confirmation, the embryos’ granulosa cells were re-extracted, the blastocyst culture medium was refreshed, and the embryos were cultured in new drops. On the afternoon of day 4, the blastocyst culture medium was replaced again and the embryos were washed three times in 25µL culture medium. These operations were efficient in removing the maternal DNA contamination. When the blastocysts met freezing criteria, they were individually vitrified and cryopreserved. Approximately 20µL of the corresponding blastocyst culture medium was collected into RNase/DNAase-free PCR tubes containing 5µL of preservation solution. The blastocysts were graded according to the Gardner score before cryopreservation (15), which assesses blastocyst expansion, inner cell mass, and trophoblast ectoderm.

### Whole genome amplification, library preparation and sequencing

A 10uL spent culture medium was pipetted from the sample preservation tube for WGA, and the sequencing library preparation was performed using the NICSInst™ (Xukang Medical Technology (suzhou) Co., Ltd) library kit (3, 9). Quality control was assessed using Qubit 3.0 (Qubit^®^ dsDNA HS Assay Kit, Thermo Fisher Scientific) and 1.5% agarose gel electrophoresis. NGS sequencing was conducted on the Illumina platform, and approximately 2M sequencing reads were obtained for each library.

### Copy number variation analysis

Data was analyzed using ChromGo™ bioinformatics software (Xukang Medical Technology (suzhou) Co., Ltd) (16). High-quality reads were counted along the genome sequence with a bin size of 1Mb, normalized for GC content and reference datasets, and analyzed with the circular binary segmentation (CBS) algorithm to identify CNV fragments. If the sequencing data do not yield a valid CNV profile, the NICS attempt is classified as a failure. Insufficient original cfDNA, whole-genome amplification failure, or poor sequencing quality can all preclude reliable CNV detection.

As previously reported 2, 17), embryos were classified into grades A, B, and C based on their probability of euploidy using the established noninvasive chromosome screening-artificial intelligence (NICS-AI) grading system. Embryos are graded as A, B, or C based on the probabilities of being euploidy as of ≥0.94, 0.7–0.94, and ≤0.7, respectively. The order of transfer follows the rule where A > B > C. For the NICS-AI system, the Random Forest machine learning algorithm was employed to construct a copy number pattern in the blastocyst culture medium that correlates with chromosomal euploidy or aneuploidy, using whole embryo CNV results as the gold standard. Eleven feature values, including the 10M-resolution CNV result, the 10M-resolution CNV result redefined by a 50% mosaicism threshold, and others, were included in the machine learning model.

### Frozen embryo thawing and second sample collection

For embryos that failed the initial NICS analysis, they were thawed and the post-thaw culture medium was collected for a second NICS testing. Thawing was performed with commercial vitrification warming solutions (Kitazato, Japan) according to the manufacturer’s four-step protocol (1 + 3 + 5 + 1 minutes). After warming, each embryo was rinsed twice in 30 µL washing micro-drops and then transferred into a 25 µL culture micro-drop for incubation.

Eight hours later, a laser-pulse (Research Instruments) was applied at the trophectoderm–opposite the inner cell mass–to induce collapse. Five minutes after collapse the blastocyst was moved to a transfer drop; 20 µL of the culture medium was aspirated into a PCR tube, using a separate Pasteur pipette for each embryo to avoid cross-contamination. The collapsed blastocyst was immediately re-vitrified with commercial vitrification freezing solutions (Kitazato, Japan) using the standard two-step procedure. This 8-h recovery interval was selected on the basis of our pilot study (**Supplementary Table 1**), which showed a high NICS amplification rate while minimizing the risk of over-hatching that is associated with longer culture time and that could compromise subsequent clinical transfer.

Similarly, morphological grading of the embryos was performed before cryopreservation. Morphological assessments were performed by the same senior embryologist before each of the two cryopreservation cycles to minimize subjective bias.

### Embryo thawing and transfer

For embryos with a successful first NICS test, patients select transfer candidates according to the NICS-AI ranking (A > B > C). Grade-A and -B embryos are recommended for transfer; if neither is available, a grade-C embryo may be used after detailed counselling and written informed consent.

When the first NICS attempt fails and no other embryos could been transferred, the cryopreserved blastocyst may be thawed and re-tested. The subsequent transfer decision follows the same hierarchy (A/B preferred; C allowed only after informed consent).

Immediately before transfer, selected blastocysts are warmed with Kitazato vitrification warming solutions (1 + 3 + 5 + 1 min protocol), rinsed twice in 30 µL wash drops, and cultured in 25 µL micro-drops. Laser-assisted hatching is then performed at a site distant from the inner cell mass where a perivitelline space is visible. Three laser pulses create a ¼–⅓ circumferential breach of the zona pellucida. Embryos are transferred 2 h after assisted hatching is completed.

### Clinical outcomes collection

The primary clinical outcome was referred as live birth rate. The HCG level was assessed 14 days post-blastocyst transfer. Clinical pregnancy was confirmed by the identification of at least one gestational sac in the uterine cavity via ultrasound at 28–30 days after transfer. Ongoing pregnancy was defined as a detectable fetal heart at week 12 of gestation. Live birth was defined as the delivery of a live infant with a gestational age exceeding 28 weeks.

### Statistical analysis

Statistical analysis was performed using the R program. Data that followed a normal distribution were presented as mean ± standard deviation (SD), and comparisons between groups were made using t-tests. Non-normally distributed data were presented as median (Q1–Q3), and the Mann-Whitney U test was used for between-group comparisons. The chi-square test was used to compare proportions or rates (%) between groups.

Multiple logistic regression analysis was used to compare clinical pregnancy rates, early miscarriage rates, ongoing pregnancy rates, and live birth rates between groups. Demographic data including female age, the number of prior miscarriage, and type of infertility were incorporated into the model to estimate the odds ratio (OR) for clinical outcomes. Statistical significance was set at *p* < 0.05 for all comparisons.

## Results

**Figure 1:**
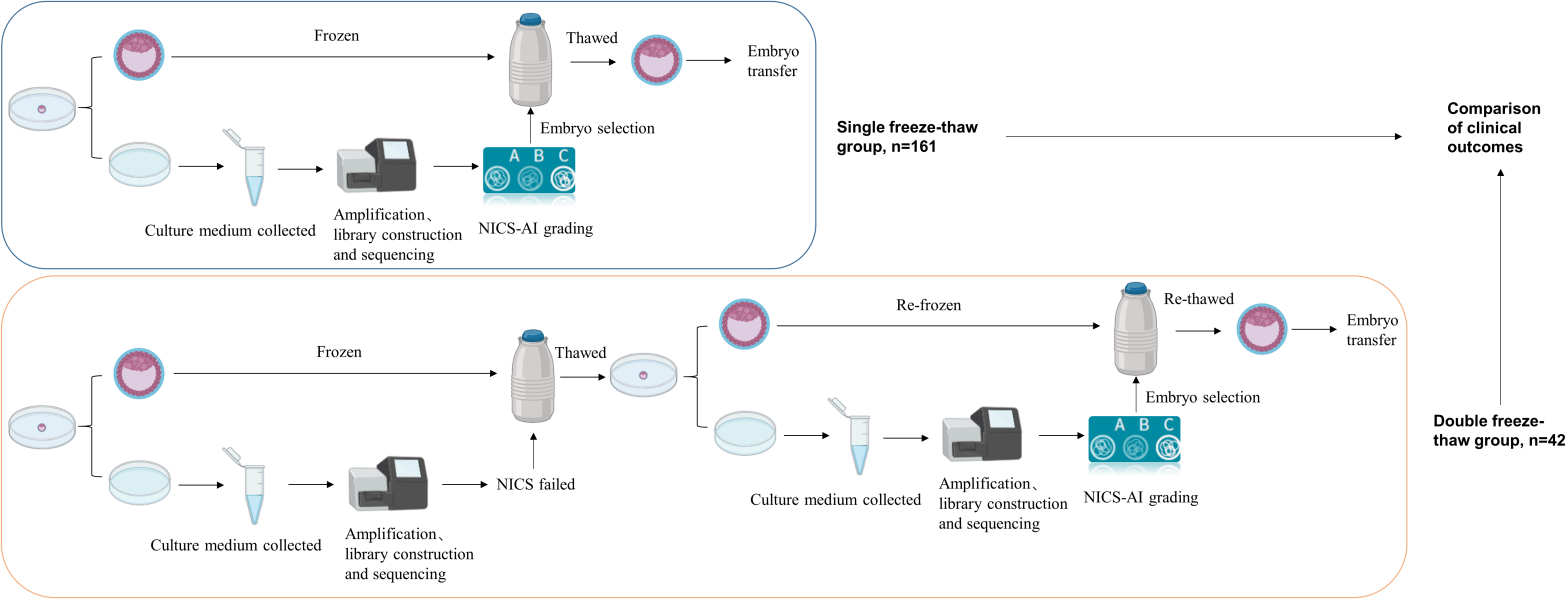
Flow chart of the clinical study. The single freeze-thaw group and the double freeze-thaw group included 161 and 42 patients for analysis, respectively. NICS, Noninvasive chromosome screening.

A total of 223 patients were included in the single freeze-thaw group, and 56 patients were included in the double freeze-thaw group based on the inclusion and exclusion criteria. Four patients from the double freeze-thaw group were excluded because they had two embryos transferred, one undergoing one freeze-thaw procedure and the other undergoing two freeze-thaw procedures. Thus, 52 patients were ultimately included in the double freeze-thaw group. The total number of patients enrolled in this test was 275. In both groups, embryos were selected for blastocyst transfer based on the NICS-AI grading system, and follow-up was continued until live birth. The study flowchart was shown in **Figure 1**.

### NICS detection success rate

**Figure 2:**
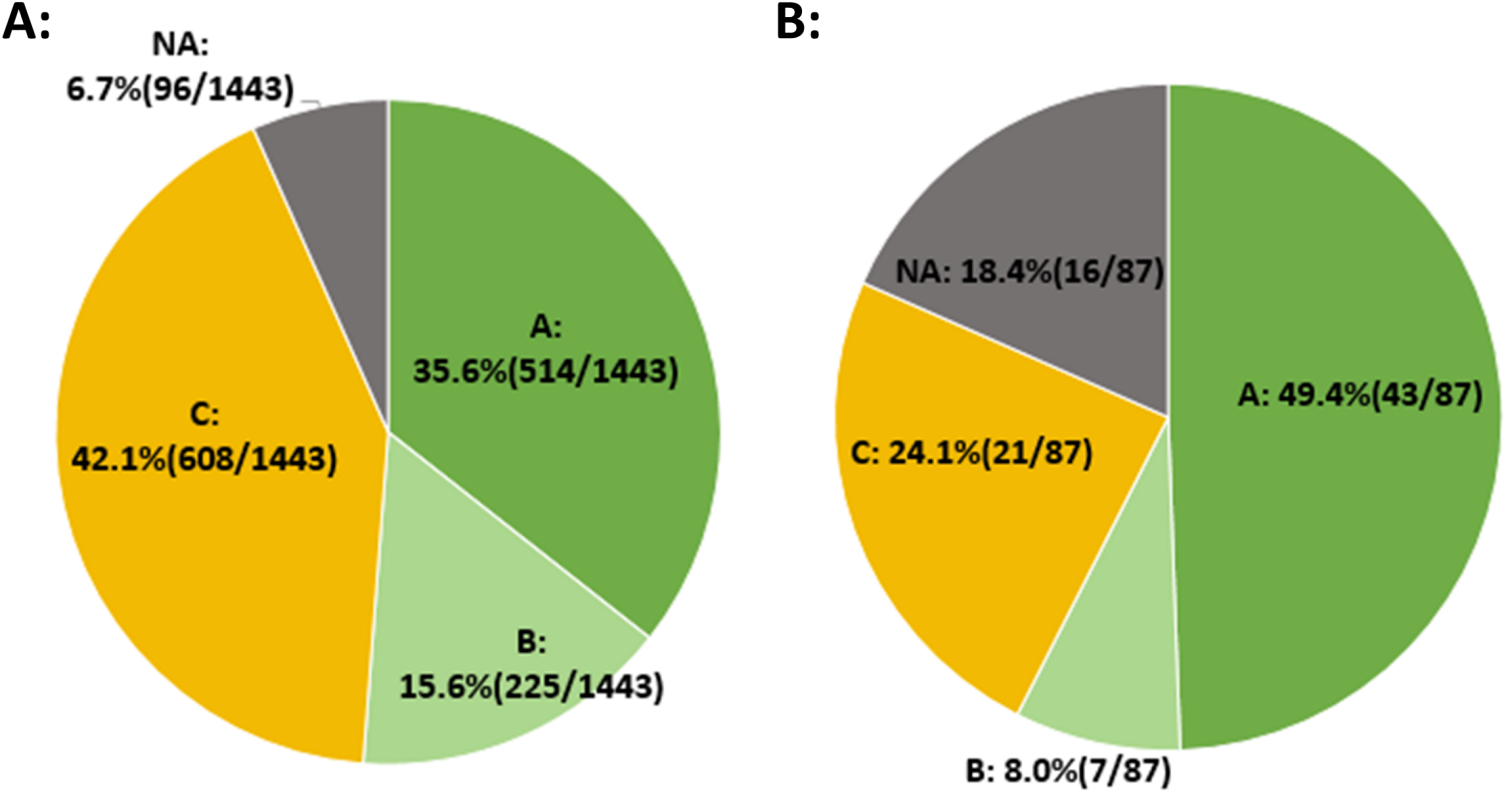
NICS detection success rate. **(A)** The initial NICS results for 1443 embryos and the proportion of embryos with different NICS-AI grades. **(B)** The second NICS results for 87 embryos that initially failed the first NICS testing.

Among the 275 patients included, a total of 1443 blastocysts were subjected to NICS testing. In the initial NICS test, 96 embryos failed, yielding a detection success rate of 93.3% (1347/1443). The proportions of blastocysts graded as A, B, and C were 35.6% (514/1443), 15.6% (225/1443), and 42.1% (608/1443), respectively (**Figure 2A**).

Of the 96 embryos that failed the initial NICS, 87 embryos were subsequently thawed and cultured for second NICS test. The results showed that the detection success rate was 81.6% (71/87). The proportions of blastocysts graded as A, B, and C were 49.4% (43/87), 8.0% (7/87), and 24.1% (21/87), respectively (**Figure 2B**).

Compared with the initial NICS results, the proportion of Grade A embryos in the second NICS results was significantly higher (35.6% vs. 49.4%, p=0.009).

### Morphological changes

To evaluate the impact of double frozen on embryo morphology, the morphological grading of embryos in the double freeze-thaw group was documented at fertilization stage, at the first freezing point, and before transfer. A total of 52 patients were included in the double freeze-thaw group, with 62 embryos thawed and transferred.

All 62 embryos were successfully thawed before transfer (100%, 62/62). Compared with the morphological grading recorded after fertilization, the expansion stage of eight embryos progressed from stage 4 to stage 5, and one embryo proliferated from stage 4 to stage 6 before the second freezing. The ICM grade of two embryos decreased from A to B, and the TE grade of two embryos decreased from B to C before the second freezing (**Table 1**, please referred to **Supplementary Figure 1** for the detailed morphology of the same embryo at three time points).

**Table 1:** Morphological changes before first freezing, second freezing, and transfer.

Morphology grading	Expansion grade			Inner cell mass (ICM)			Trophectoderm (TE)		
	4	5	6	A	B	C	A	B	C
Before first freezing	62	0	0	9	51	2	10	34	18
Before second freezing	53	8	1	7	53	2	10	32	20
Before transfer	11	44	7	7	53	2	10	32	20

There were no changes in the ICM and TE grades before the second freezing to that of at transfer period. However, 42 embryos expanded from stage 4 to stage 5 or 6. This phenonmenon is consistent with the stipulation of the standard operation practive (SOP) that indicates *in vitro* embryos must reach to stage 5 before transfer.

### Patients characteristics

Ten and sixty-two patients were excluded from the double freeze–thaw and single freeze–thaw groups, respectively, because they underwent double-embryo transfer. There were 42 patients in the double freeze-thaw group and 161 patients in the single freeze-thaw group (**Figure 1**).

The patients’ baseline characteristics and details of the transferred embryos are shown in **Table 2**. There was a significant difference between the two groups in terms of female age, the number of prior miscarriage and type of infertility (p<0.05). The differences in BMI, male age, duration of infertility, the number of prior failed transfer, IVF indication and AMH between the two groups were not statistically significant (p>0.05). Between the single and double freeze–thaw groups, no differences were observed in NICS-AI grade, quality grade, or blastocyst formation day of the transferred embryos (p>0.05).

**Table 2:** The basic characteristics of the single freeze–thaw group and the double freeze–thaw group.

Variable	Single freeze-thaw group (n = 161)	Double freeze-thaw group (n = 42)	P value
Female age (mean ± SD)	33.52 ± 4.61	31.62 ± 4.42	0.018
Female BMI (mean ± SD)	23.33 ± 3.51	22.81 ± 3.75	0.396
Male age (mean ± SD)	34.32 ± 5.25	33.24 ± 4.72	0.225
Male BMI (mean ± SD)	26.94 ± 3.93	27.35 ± 4.09	0.548
Duration of infertility, M (Q_1_, Q_3_)	2.00 (1.00, 4.00)	2.00 (1.00, 4.00)	0.303
No. of prior failed transfer, n (%)			0.364
0	123 (76.40)	36 (85.71)	
1-2	30 (18.63)	4 (9.52)	
≥3	8 (4.97)	2 (4.76)	
No. of prior miscarriage, n (%)			0.010
0	68 (42.24)	28 (66.67)	
1	36 (22.36)	8 (19.05)	
≥2	57 (35.40)	6 (14.29)	
IVF indication, n (%)			0.181
Male factor	6 (3.73)	4 (9.52)	
Female factor	144 (89.44)	34 (80.95)	
Both	11 (6.83)	4 (9.52)	
Type of infertility, n (%)			0.009
Primary infertility	25 (15.53)	14 (33.33)	
Secondary infertility	136 (84.47)	28 (66.67)	
AMH, M (Q_1_, Q_3_)	3.25 (1.96, 4.66)	4.00 (2.49, 6.48)	0.059
Method of fertilization, n (%)			0.136
ICSI	17 (10.56)	8 (19.05)	
IVF	144 (89.44)	34 (80.95)	
No. of blastocysts, M (Q_1_, Q_3_)	4.00 (3.00, 7.00)	5.00 (3.00, 8.75)	0.130
NICS-AI grading, n (%)			0.062
A/B	124 (77.02)	30 (71.43)	
C	32 (19.88)	7 (16.67)	
NA	5 (3.11)	5 (11.90)	
Embryonic days, n (%)			0.695
Day 5	86 (53.42)	25 (59.52)	
Day 6	69 (42.86)	15 (35.71)	
Day 7	6 (3.73)	2 (4.76)	
Quality grade, n (%)			0.265
Good (AA, AB, BA)	36 (22.36)	11 (26.19)	
Fair (BB)	61 (37.89)	20 (47.62)	
Poor (AC, CA, BC, CB)	64 (39.75)	11 (26.19)	

### Clinical outcomes

Clinical outcomes in the single and double freeze–thaw groups were analyzed, and as shown in **Supplementary Table 2**. No significant differences were observed in clinical pregnancy rate (56.52% vs. 57.14%, p=0.942), early miscarriage rate (21.98% vs. 25.00%, p=0.753), ongoing pregnancy rate (44.10% vs. 42.86%, p=0.885), and live birth rate (42.86% vs. 42.86%, p=1.000).

Since the patients in the single freeze–thaw group and double freeze–thaw group were statistically different (p<0.05) in terms of female age, the number of prior miscarriage and type of infertility, these variables were taken as independent variables and included in the binary logistic regression analyses. The results showed that there were still no significant differences in the clinical pregnancy rate, early miscarriage rate, ongoing pregnancy rate, and live birth rate in single freeze–thaw group and double freeze–thaw group (adjusted p value > 0.05) (**Table 3**).

**Table 3:** Logistic regression analysis compared the clinical outcomes of the single freeze–thaw group and the double freeze–thaw group.

Clinical outcomes	Single freeze-thaw group (n = 161)	Double freeze-thaw group (n = 42)	Adjusted P value*	Adjusted OR (95% CI)*
Clinical pregnancy rate	56.52% (91/161)	57.14% (24/42)	0.785	0.90 (0.44 ~ 1.86)
Early miscarriage rate	22.00% (20/91)	25.00% (6/24)	0.528	1.43 (0.47 ~ 4.36)
Ongoing pregnancy rate	44.10% (71/161)	42.86% (18/42)	0.516	0.79 (0.38 ~ 1.62)
Live birth rate	42.86% (69/161)	42.86% (18/42)	0.736	0.88 (0.43 ~ 1.82)

*Logistic regression analysis adjusted for the female age, No. of prior miscarriage and type of infertility.

## Discussion

In this study, the embryos with NA results over the first NICS were thawed and cultured for 8 hours, after which the culture medium was collected for the second NICS test. The detection success rate was 81.6% (71/87), with 57.4% of the embryos are allowed to be transferred follow the stipulation of SOP. The clinical pregnancy rate, early miscarriage rate, ongoing pregnancy rate, and live birth rate were similar between the double freeze-thaw group and single freeze-thaw group. Thus, re-testing embryos that initially yield not available NICS results after thawing maximized embryo utilization, and patients who proceed with transfer based on the second NICS result can still achieve favorable clinical outcomes. Moreover, the proportion of Grade A embryos in the double freeze-thaw group was higher than that in single freeze-thaw group (49.4% vs. 35.6%, p=0.009). This may be due to the higher rate of euploidy in embryos that initially failed the NICS test. Nakhuda et al. reported that patients who underwent transfer of embryos that failed niPGT had the highest ongoing pregnancy rates compared with those who had transfers of euploid and aneuploid embryos (66.7% vs. 57.3% vs. 41.2%, respectively) (18). The study by Huang et al. demonstrated that patients who transferred embryos with lower cfDNA levels achieved higher live birth rates compared to those with higher cfDNA levels (37.56% vs. 23.04%, p < 0.001) (19). This may be attributed to better embryo quality and more compact cellular structure, leading to reduced DNA release into the culture medium. Therefore, embryos that initially failed NICS testing may also have a higher potential for implantation. By thawing these embryos and collecting the culture medium for second NICS testing, the chromosomal status of 81.6% (71/87) of the embryos was determined. Transferring embryos based on the results of the second NICS test achieved clinical outcomes comparable to those of embryos that initially succeeded in NICS.

Koch et al. demonstrated that no significant differences was observed in clinical pregnancy rate and live birth rate between double freeze-thaw embryos and single freeze-thaw embryos (20). However, other studies have shown that double freezing and thawing may impair the developmental potential of blastocysts and reduce live birth rates (21, 22). In our study, the expansion degree and ICM and TE morphology grades of embryos in the double freeze-thaw group were recorded both before and after cryopreservation and thawing. After 8 hours of culture following thawing, the morphology of the ICM and TE of the embryos remained essentially unchanged. Nine embryos progressed from stage 4 to stage 5 or 6. Most embryos were cultured to stage 5 before transfer, which was in line with the routine practice of our center, where embryos are generally cultured to stage 5 for transfer. The survival rate of embryos that underwent double freezing was 100% (62/62). Regalado et al. also reported that double freezing may not affect embryo survival (23). Therefore, performing a second NICS test on thawed embryos maybea viable option following initial NICS failure, and embryo transfer can be guided by the second NICS results. More future data are required to directly compare the clinical outcomes of using or not using the NICS protocol during frozen embryo cycles.

Moreover, this study offers alternative solutions for embryos that fail PGT-A testing. For embryos that initially failed PGT-A or were identified as complex mosaicism, when patients have no other euploid embryos available for transfer, clinicians may choose to thaw and re-biopsy these embryos for a second PGT-A test to avoid embryo wastage. Zhou et al. performed a second biopsy and vitrified the embryos with complex mosaicism detected by PGT-A. The results showed that the success rate of thawing and re-warming of these embryos was 100%, with a euploid rate of 61.6% and an ongoing pregnancy rate of 38.9% for patients (24). However, PGT-A required embryo biopsy and even a second biopsy, which not only required professional skills but also may affect embryo implantation and even offspring development (25, 26). NICS sampling is noninvasive. Several studies have shown that culture medium testing of mosaic embryos post-thaw yields NICS results that more accurately reflect the overall condition of the embryos. Huang et al. found that, using donated mosaic and aneuploid embryos, NICS better reflected the chromosomal status of embryos than PGT-A (4). Li et al. thawed 41 mosaic embryos, and 85.36% (35/41) of whole embryo were euploid, with 82.85% (29/35) of the corresponding NICS results also being euploid (27). That is, NICS can give 70% of embryos with mosaic results from PGT-A a second chance for transfer, thus avoiding the waste of mosaic embryos. Therefore, NICS could serve as an alternative solutions for embryos that initially failed PGT-A or were identified as complex mosaicism, allowing embryo selection and transfer to be guided by the NICS result.

In addition to detection failure, maternal contamination is a bottleneck for NICS. At present, the proportion of maternal contamination in the culture medium can already be identified (28). Studies have shown that more than half (95/191) of embryos may be affected by maternal contamination, with nearly 50% (45/95) of these embryos having a maternal contamination proportion of over 40% (29). Huang et al. have shown that with an appropriate sampling protocol, maternal contamination can be reduced to 5–6.8%. The cumulus cells were removed at the oocyte stage, and the embryos were washed and transferred to fresh medium on both days 3 and 4 to minimize any residual granulosa-cell contamination (16). If an embryo is detected with a high proportion of maternal contamination, clinicians and patients may be concerned about the increased risk of transfer. In such cases, the frozen embryos can be thawed to obtain the culture medium, which is free of maternal contamination, for NICS. Xie et al. found that the proportion of embryos with maternal contamination in the culture medium after thawing was lower compared to that in fresh culture (19.2% vs. 35.8%, p=0.033) (30). This may be related to the source of maternal contamination. The primary source of maternal contamination in the culture medium is the attachment of granulosa cells. Multiple medium changes and washings during the freezing and thawing process reduce the likelihood of granulosa cell attachment. Additionally, maternal cells, such as polar bodies or free DNA from follicular fluid cell degradation, may be displaced and diluted during the osmotic changes of freezing and thawing, thereby reducing maternal DNA interference.

For patients with a history of recurrent implantation failure or miscarriage who have cryopreserved embryos conceived by conventional IVF (c-IVF), PGT-A is precluded because of the possible contamination from cumulus cells. In these cases, only morphological grading can be applied for embryo selection, with no information on ploidy status. In the future, thawing such embryos and performing NICS on the spent blastocyst culture medium mayebe anoption. Indeed, the study has shown that NICS results obtained from c-IVF-derived blastocysts are comparable to those from ICSI-derived embryos, and their diagnostic accuracy is equivalent to that of trophectoderm biopsy (30).This study also has its limitations. First, the study utilized the culture medium from embryos that initially failed NICS testing, rather than from all embryos. These embryos that failed NICS initially tend to have better morphology and a higher probability of being euploid, which may be more conducive to achieving better pregnancy outcomes for patients. The double freeze–thaw group in this study was small; larger samples are needed to confirm these findings. Second, to ensure the survival rate of thawed embryos, the embryo culture volume was 25μL. However, the detection success rate still requires further improvement, for example, by using a 10μL-culture volume for embryo thawing and culturing. Ardestani et al. thawed and cultured frozen embryos in a 10μL-volume for 8 hours and found that the detection success rate of NICS could reach 100% (22/22) in embryos (14). Finally, patients who were transferred double freeze-thaw embryos still require long-term follow-up of neonatal outcomes to verify current finding.

Novel approaches are being developed currently by means to reduce the need for double freezing and thawing. The rapid NICS can be achieved via leveraging the short sequencing time of third-generation sequencing platforms. This approach would allow embryos to undergo NICS after the first thawing and culturing without the need for a second freeze-thaw cycle, thereby reducing operational costs and potential harm to the embryos. Consequently, patients can proceed with embryo transfer more quickly.

In conclusion, this study suggested that for embryos that initially failed NICS testing, a second NICS could be performed after thawing the frozen embryos. This approach was safe and increased embryo utilization. Patients undergoing transfer of embryos selected by second NICS results achieved clinical outcomes similar to those receiving embryos with a single freeze-thaw cycle.

## Data Availability

The original contributions presented in the study are included in the article/**Supplementary Material**. Further inquiries can be directed to the corresponding authors.

## Supplementary material

Supplementary Figure 1 Morphological grading of the same embryo before and after the two freeze-thaw procedures.

## References

[1] Xu J Fang R Chen L Chen D Xiao JP Yang W. Noninvasive chromosome screening of human embryos by genome sequencing of embryo culture medium for *in vitro* fertilization. Proc Natl Acad Sci United States America. (2016) 113:11907–12. doi: 10.1073/pnas.1613294113, PMID: 27688762 PMC5081593

[2] Li X Yao Y Zhao D Chang X Li Y Lin H. Clinical outcomes of single blastocyst transfer with machine learning guided noninvasive chromosome screening grading system in infertile patients. Reprod Biol Endocrinol. (2024) 22:61. doi: 10.1186/s12958-024-01231-9, PMID: 38783347 PMC11112939

[3] Chen L Sun Q Xu J Fu H Liu Y Yao Y. A non-invasive chromosome screening strategy for prioritizing *in vitro* fertilization embryos for implantation. Front Cell Dev Biol. (2021) 9:708322. doi: 10.3389/fcell.2021.708322, PMID: 34434931 PMC8380813

[4] Huang L Bogale B Tang Y Lu S Xie XS Racowsky C. Noninvasive preimplantation genetic testing for aneuploidy in spent medium may be more reliable than trophectoderm biopsy. Proceedings of the national academy of sciences of the United States of America. (2019) 116:14105–12. doi: 10.1073/pnas.1907472116, PMID: 31235575 PMC6628824

[5] Xi H Qiu L Yao Y Luo L Sui L Fu Y. Noninvasive chromosome screening for evaluating the clinical outcomes of patients with recurrent pregnancy loss or repeated implantation failure. Front Endocrinol (Lausanne). (2022) 13:896357. doi: 10.3389/fendo.2022.896357, PMID: 35800428 PMC9253989

[6] Rubio C Navarro-Sánchez L García-Pascual CM Ocali O Cimadomo D Venier W. Multicenter prospective study of concordance between embryonic cell-free DNA and trophectoderm biopsies from 1301 human blastocysts. Am J obstetrics gynecology. (2020) 223:751.e751–751.e713. doi: 10.1016/j.ajog.2020.04.035, PMID: 32470458

[7] Kuznyetsov V Madjunkova S Antes R Abramov R Motamedi G Ibarrientos Z. Evaluation of a novel non-invasive preimplantation genetic screening approach. PloS One. (2018) 13:e0197262. doi: 10.1371/journal.pone.0197262, PMID: 29746572 PMC5944986

[8] Yeung QSY Zhang YX Chung JPW Lui WT Kwok YKY Gui B. A prospective study of non-invasive preimplantation genetic testing for aneuploidies (NiPGT-A) using next-generation sequencing (NGS) on spent culture media (SCM). J assisted Reprod Genet. (2019) 36:1609–21. doi: 10.1007/s10815-019-01517-7, PMID: 31292818 PMC6707994

[9] Jiao J Shi B Sagnelli M Yang D Yao Y Li W. Minimally invasive preimplantation genetic testing using blastocyst culture medium. Hum Reprod. (2019) 34:1369–79. doi: 10.1093/humrep/dez075, PMID: 31251795

[10] Parriego M Coll L Vidal F Boada M Devesa M Coroleu B. Inconclusive results in preimplantation genetic testing: go for a second biopsy? Gynecological endocrinology: Off J Int Soc Gynecological Endocrinology. (2019) 35:90–2. doi: 10.1080/09513590.2018.1497153, PMID: 30182774

[11] Cimadomo D Rienzi L Romanelli V Alviggi E Levi-Setti PE Albani E. Inconclusive chromosomal assessment after blastocyst biopsy: prevalence, causative factors and outcomes after re-biopsy and re-vitrification. A multicenter experience. Hum reproduction. (2018) 33:1839–46. doi: 10.1093/humrep/dey282, PMID: 30239718

[12] De Vos A Van Landuyt L De Rycke M Verdyck P Verheyen G Buysse A. Multiple vitrification-warming and biopsy procedures on human embryos: clinical outcome and neonatal follow-up of children. Hum reproduction. (2020) 35:2488–96. doi: 10.1093/humrep/deaa236, PMID: 33047114

[13] Chen R Tang N Du H Yao Y Zou Y Wang J. Clinical application of noninvasive chromosomal screening for elective single-blastocyst transfer in frozen-thawed cycles. J Trans Med. (2022) 20:553. doi: 10.1186/s12967-022-03640-z, PMID: 36463184 PMC9719190

[14] Ardestani G Banti M García-Pascual CM Navarro-Sánchez L Van Zyl E Castellón JA. Culture time to optimize embryo cell-free DNA (cfDNA) analysis for frozen-thawed blastocysts undergoing non-invasive preimplantation genetic testing for aneuploidy (niPGT-A). Fertility sterility. (2024) 122:465–73. doi: 10.1016/j.fertnstert.2024.04.037, PMID: 38718960

[15] Gardner DK Lane M Stevens J Schlenker T Schoolcraft WB. Blastocyst score affects implantation and pregnancy outcome: towards a single blastocyst transfer. Fertility sterility. (2000) 73:1155–8. doi: 10.1016/s0015-0282(00)00518-5, PMID: 10856474

[16] Huang J Yao Y Jia J Zhu X Ma J Wang J. Chromosome screening of human preimplantation embryos by using spent culture medium: sample collection and chromosomal ploidy analysis. J visualized experiments. (2021) 7:175. doi: 10.3791/62619, PMID: 34570097

[17] Chen L Li W Liu Y Peng Z Cai L Zhang N. Non-invasive embryo selection strategy for clinical IVF to avoid wastage of potentially competent embryos. Reprod biomedicine Online. (2022) 45:26–34. doi: 10.1016/j.rbmo.2022.03.006, PMID: 35537927

[18] Nakhuda G Rodriguez S Tormasi S Welch C. A pilot study to investigate the clinically predictive values of copy number variations detected by next generation sequencing of cell free DNA in spent culture media. Fertility sterility. (2024) 122:42–51. doi: 10.1016/j.fertnstert.2024.02.030, PMID: 38382698

[19] Huang J Yao Y Jia J Wang Z Shi X Li Y. Library concentration of cell-free DNA in spent culture medium: a potential indicator for clinical outcomes of blastocyst transfer. Reprod biomedicine Online. (2024) 51:104752. doi: 10.1016/j.rbmo.2024.104752, PMID: 40544577

[20] Koch J Costello MF Chapman MG Kilani S. Twice-frozen embryos are no detriment to pregnancy success: a retrospective comparative study. Fertility sterility. (2011) 96:58–62. doi: 10.1016/j.fertnstert.2011.04.034, PMID: 21570070

[21] Wang M Zhou J Long R Li Y Gao L Mao R. Recryopreservation impairs blastocyst implantation potential via activated endoplasmic reticulum stress pathway and induced apoptosis. MedComm. (2024) 5:e689. doi: 10.1002/mco2.689, PMID: 39156765 PMC11329749

[22] Wang M Jiang J Xi Q Li D Ren X Li Z. Repeated cryopreservation process impairs embryo implantation potential but does not affect neonatal outcomes. Reprod biomedicine online. (2021) 42:75–82. doi: 10.1016/j.rbmo.2020.11.007, PMID: 33309388

[23] López Regalado ML Clavero A Gonzalvo MC Serrano M Martínez L Mozas J. Cumulative live birth rate after two single frozen embryo transfers (eSFET) versus a double frozen embryo transfer (DFET) with cleavage stage embryos: a retrospective cohort study. J assisted Reprod Genet. (2014) 31:1621–7. doi: 10.1007/s10815-014-0346-5, PMID: 25267163 PMC4250464

[24] Zhou S Xie P Zhang S Hu L Luo K Gong F. Complex mosaic blastocysts after preimplantation genetic testing: prevalence and outcomes after re-biopsy and re-vitrification. Reprod biomedicine Online. (2021) .43:215–22. doi: 10.1016/j.rbmo.2021.04.006, PMID: 34193357

[25] Scott RT Ferry K Su J Tao X Scott K Treff NR. Comprehensive chromosome screening is highly predictive of the reproductive potential of human embryos: a prospective, blinded, nonselection study. Fertility sterility. (2012) 97:870–5. doi: 10.1016/j.fertnstert.2012.01.104, PMID: 22305103

[26] Zhang WY von Versen-Höynck F Kapphahn KI Fleischmann RR Zhao Q Baker VL. Maternal and neonatal outcomes associated with trophectoderm biopsy. Fertility sterility. (2019) 112:283–290.e282. doi: 10.1016/j.fertnstert.2019.03.033, PMID: 31103283 PMC6527329

[27] Li X Hao Y Chen D Ji D Zhu W Zhu X. Non-invasive preimplantation genetic testing for putative mosaic blastocysts: a pilot study. Hum reproduction. (2021) 36:2020–34. doi: 10.1093/humrep/deab080, PMID: 33974705

[28] Dong Y Liu D Zou Y Wan C Chen C Dong M. Preimplantation genetic testing for human blastocysts with potential parental contamination using a quantitative parental contamination test (qPCT): an evidence-based study. Reprod biomedicine online. (2023) 46:69–79. doi: 10.1016/j.rbmo.2022.08.103, PMID: 36257886

[29] Chen Y Gao Y Jia J Chang L Liu P Qiao J. DNA methylome reveals cellular origin of cell-free DNA in spent medium of human preimplantation embryos. J Clin Invest. (2021) 131:e146051. doi: 10.1172/jci146051, PMID: 34128477 PMC8203451

[30] Xie P Zhang S Gu Y Jiang B Hu L Tan YQ. Non-invasive preimplantation genetic testing for conventional IVF blastocysts. J Trans Med. (2022) 20:396. doi: 10.1186/s12967-022-03596-0, PMID: 36058949 PMC9441092

