# Supplementary material for: Clinical outcomes of frozen-thawed blastocysts with twice noninvasive chromosome screenings
Source: Front Endocrinol (Lausanne). 2025 Oct 30;16:1699690. doi: 10.3389/fendo.2025.1699690 (PMC12611674; doi:10.3389/fendo.2025.1699690)
Supplement: Supplementary file 2 [file Table1.docx]

**Supplementary Table 1. NICS success rate of embryo thawing culture medium at different sampling times.**

| **Groups** | **Sample size** | **NICS success rate** | **Concordance (versus whole embryo)** |
| --- | --- | --- | --- |
| **4h** | **5** | **80%（4/5）** | **25%（1/4）** |
| **6h** | **11** | **90.9%(10/11)** | **60%（6/10）** |
| **8h** | **24** | **95.8%（23/24）** | **91.3%（21/23）** |

**Supplementary Table 2. Comparison of clinical outcomes between the single freeze–thaw group and the double freeze–thaw group.**

| **Clinical outcomes** | **Single freeze-thaw group**  **(n = 161)** | **Double freeze-thaw group**  **(n = 42)** | **P value** |
| --- | --- | --- | --- |
| **Clinical pregnancy rate** | **56.52%(91/161)** | **57.14%(24/42)** | **0.942** |
| **Early miscarriage rate** | **21.98%(20/91)** | **25.00%(6/24)** | **0.753** |
| **Ongoing pregnancy rate** | **44.10%(71/161)** | **42.86%(18/42)** | **0.885** |
| **Live birth rate** | **42.86%(69/161)** | **42.86%(18/42)** | **1.000** |
